# 

**Published:** 1822-05

**Authors:** 


### MONTHLY CATALOGUE OF MEDICAL BOOKS.

The Way to preserve Health and attain Longevity, with a familiar Treatise on Domestic Medicine, pointing out the Diseases of Men, Women, and Children, in all Climates; with Prescriptions in English, and the proper Doses of Medicine. By Robert Thomas, M.D. &c. &c. 1 vol. 8vo. 15s.

Essays, Physiological and Practical. By James Carson, M.D. Physician in Liverpool. 8vo. 3s.

Medicamina Officinalia, seu Pharmacopœiæ Londinensis, Index Methodicus. Cura F. A. Macann, M.D. 18mo.

A Pharmaceutical Guide: in two Parts.—Part I. A Latin Grammar, in which all the Rules are illustrated by Examples selected from the London Pharmacopœia. —Part II. An Interlineary Translation of such Formulæ in the London Pharmacopœia as have been found difficult to be comprehended by some young Medical Students. To which is prefixed, a Vocabulary of Words most frequently employed in Prescriptions; with Examples of their Use. By the Author of the Student's Manual. 12mo. 5s. 6d.

The New Medico-Chirurgical Pharmacopœia: for the Use of Surgeons and Surgeon-Apothecaries. By a Member of the Colleges of Surgeons of London and Edinburgh. 12mo. 5s.

Two Additional Numbers of the Medical Spectator: in one of which the Author advances a Claim to the first Promulgation of some modern Improvements in the Healing Art; the other contains an Essay on Congenital Dyspepsy. 1s. 6d.

### NOTICES TO CORRESPONDENTS.

*We are obliged to* Mr. Purton *for his letter, and the very flattering expressions it contains. It is a singular circumstance that, at the time we received* Mr. Purton's *letter, we had just met with a striking case of what he had alluded to, which was nigh proving destructive to the patient.* Mr. Purton *is correct in his supposition; and we have included the modification he mentions as one which produces abortion, under the head of "Peculiarities of the Ovum," in our classification of Miscarriages. We have not received the work mentioned in his letter.*

*We trust we have succeeded in giving* Mr. Batty *satisfaction.*

Mr. Bush *has our thanks for his letter of the 3d of April. An association, like the one he suggests, would be most useful even in London, where we are pestered both with* respectable *irregulars, and regulars that are not* respectable.

Messrs. Yeatman *and* Norman *the earliest opportunity.*

*Our Correspondents are requested to observe, that Communications, and Books for Review, should be addressed* "To the Editor of the London Medical and Physical Journal, care of Mr. Souter, No. 73, St. Paul's Church-Yard, London."

*The Proprietors beg to acquaint the Medical Profession, that they have a few Copies remaining of the* GENERAL INDEX, *which comprises an Analytical Table of Contents, from* Volume One to Forty *inclusive; carefully arranged in Alphabetical Order, with References to the whole of the cited Authorities, under their nominal Characters, &c. and forming an indispensable* Appendix *to the Journal; in one large* 8vo. *volume, price* 21s.

